# An Encyclopedia of Practical Medicine, Based on Bacteriology, Etc.

**Published:** 1890-11

**Authors:** 


AN ENCYCLOPEDIA OF PRACTICAL MEDICINE, BASED UPON BACTERIOLOGY. One large octavo volume, 1456 pages, 800 illustrations. Price $3.25, sent by express, prepaid. R. R. Russell, Publisher, 59 East Ninth street, New York. 1890.

The present volume is the first American work upon bacteriology. Its aim is to give a practical elucidation of the germ theory of disease.

It is illustrated with accurate microscopical, photographical wood-cuts of all known pathogenic microbes.

It gives a complete working synopsis of bacteriological investigations with reference to all microbes as the cause of the more important diseases, such as:

1. Diseases whose bacterial cause is determined with comparative certainty: Anthrax, caused by Bacillus anthracis. Aphtha, caused by Oidium albicans. Cholera, caused by comma bacillus. Erysipelas, caused by Streptococcus erysipelatosus. Gonorrhea, caused by the gonococcus. Leprosy, caused by the lepra bacillus. Malarial fever, caused by bacillus malariæ. Meningitis (epidemic, cerebro-spinal), caused by Diplococcus lanceolatus. Pertussis, caused by a bacillus. Pneumonia, caused by Diplococcus pneumoniæ. Purpura, caused by Monas hæmorrhagica. Pyæmia, caused by Streptococcus pyogenès. Relapsing fever, caused by a spirillum. Tetanus, caused by a "pin-head" bacillus. Tuberculosis, caused by the tubercle bacillus. Typhoid fever, by Bacillus typhosus. Typhus fever, caused by a bacillus.

2. Bacterial diseases, whose exciting cause has not been so perfectly determined: Carcinoma, dengue, diphtheria, dysentery, gangrene, glanders, measles, parotitis, rabies, rheumatism, rötheln, scarlatina, syphilis, yellow fever, croup.

All catarrhal diseases, such as bronchitis, conjunctivitis, diarrhœa, etc., are of bacterial origin, and various bacteria are engaged as causative factors in different varieties of these several diseases. These have been isolated with varying degrees of certainty, and are genuine pathogenic microbes.

The streptococcus of diphtheria is one of the most remarkable of all micro-organisms—a bacterium capable of inducing the formation of a false membrane, with bacterial secreta, a ptomaine of the most paralyzing character.

The mode of culture, and the best bactericides for their sterilization and complete annihilation. The cadaveric alkaloids or ptomaines excreted by each microbe in process of sporulation, are given in tabulated form.

It is a practice of medicine from a microbial standpoint.

In addition there is a chapter on Sanitary Science, illustrated, well adapted for Health Boards. B.

